# Fluoroquinolone resistance without fluoroquinolone use; the genie is truly out of the bottle

**DOI:** 10.3389/fmicb.2026.1842637

**Published:** 2026-08-18

**Authors:** Evgeni Sokurenko, Miklos Fuzi

**Affiliations:** 1 Department of Microbiology, University of Washington School of Medicine, Seattle, WA, United States; 2 Independent Researcher, Seattle, WA, United States

**Keywords:** carriage without antibiotics, clone, fitness, multidrug-resistant pathogen, QRDR mutations

## Abstract

The global dissemination of multidrug-resistant (MDR) bacteria is not merely a clinical byproduct of antibiotic overuse, but an evolutionary transition driven by highly successful, human-adapted clonal lineages. While MDR phenotypes are frequently associated with resistance to various drug classes, FQR—mediated by specific mutations in the Quinolone Resistance-Determining Regions (QRDR) of the chromosomal GyrA and ParC genes—stands out as a primary driver of clonal success. Contrary to the traditional “fitness cost” paradigm of antibiotic resistance, recent evidence suggests long-term commensal colonization of healthy individuals by MDR bacteria even in environments devoid of recent antibiotic use. This review analyzes the literature supporting the role of certain QRDR mutations in the commensal carriage of *Escherichia coli*, *Staphylococcus aureus*, and other opportunistic human pathogens. We propose, among other explanations, that the metabolic surplus generated by some QRDR mutations offsets the cost of carrying extensive resistance gene cargoes, allowing MDR clones to outcompete susceptible wild-type strains in the community in the absence of antibiotic pressure. Consequently, public health strategies must evolve beyond antibiotic stewardship alone, toward preventing community transmission of MDR strains and effective decolonization of colonized individuals—the main reservoir of clinically relevant antibiotic resistance.

## 1 Introduction

The crisis of antibiotic resistance is often framed as a direct cause-and-effect response to selective pressure: the more we use antibiotics, the more resistance we see. While this remains true, it fails to explain the persistent, sometimes decades-long dominance of MDR strains from certain genetic lineages (clones) in the community, where antibiotic pressure is often negligible (Huang et al., 2006; David and Daum, 2010; Gurnee et al., 2015; Spellberg and Doi, 2015; Neut, 2021; Antimicrobial Resistance Collaborators, 2022; Tchesnokova et al., 2023; Cuny et al., 2024; Suzhaeva et al., 2026; Tano et al., 2026).

The dissemination of most MDR bacterial pathogens is fundamentally clonal (Woodford et al., 2011; Klemm et al., 2018; Teng et al., 2023), meaning that a few “super-clones” account for the vast majority of global infections. Identifying the biological basis of this clonal success is therefore of primary importance. It has been shown that the MDR phenotype is largely associated with FQR, and specifically with the carriage of generally three mutations in the so-called quinolone resistance-determining regions (QRDR) of the main targets of fluoroquinolones—type II topoisomerases: DNA gyrase subunit A (GyrA) and topoisomerase IV (ParC)—which are essential for managing DNA supercoiling, transcription, and replication (Hooper, 2001; Redgrave et al., 2014; Fuzi, 2016; Fuzi et al., 2017; Fuzi et al., 2020; Fuzi and Sokurenko, 2023; Choudhury et al., 2024; Kherroubi et al., 2024; Fuzi, 2025; Nehru et al., 2024). Beyond QRDR mutations, various genetic attributes can contribute to the FQR phenotype in bacteria, including overexpression of efflux pumps, altered outer membrane permeability via mutations in the corresponding chromosomal genes, or acquisition of plasmid-mediated quinolone resistance (PMQR) genes encoding target protection proteins, FQ modification enzymes, or additional efflux pumps (Van der Putten et al., 2019; Elbehiry et al., 2026). These mechanisms, however, do not typically confer the high-level resistance seen in natural FQR strains with elevated minimum inhibitory concentrations. They usually impose either a high fitness cost (as with efflux pump overexpression) or provide a secondary, add-on contribution to resistance (as with plasmid-borne genes), and by themselves, fail to protect bacteria against therapeutic drug levels as achieved by QRDR mutations (Marcusson et al., 2009; Van der Putten et al., 2019). Thus, the presence of QRDR mutations is the most typical attribute of all clinically significant FQR clones across different species, in contrast to resistance-associated plasmids, which are a less stable characteristic (Toth et al., 2014; Zhao et al., 2015; Tano et al., 2026).

In this review we present evidence showing that available data strongly support the association of QRDR mutations with many international MDR bacterial clones and their successful dissemination not only in the healthcare setting but also in the community as commensal colonizers of healthy people. We then discuss the possible mechanisms behind the fitness advantage of MDR clonal lineages, directly or indirectly linked to the presence of QRDR mutations. Finally, we briefly review endeavors aimed at minimizing the community spread of MDR international bacterial clones through broad surveillance and active decolonization strategies.

This review focuses primarily on the two bacterial species most commonly isolated in clinical microbiology laboratories—*Escherichia coli* and *Staphylococcus aureus*—with attention to extraintestinal pathogenic *E. coli* (ExPEC) and methicillin-resistant *S. aureus* (MRSA). Within these species, QRDR mutations are found across a wide variety of strains.

## 2 Carriage of MDR pathogens in healthy individuals

### 2.1 E. coli

The MDR phenotype among ExPEC strains is currently dominated by a few globally disseminated FQR clonal lineages, among which the most prominent are subclone H30 of ST131 (ST131-H30, also known as ST131 clade C) and the more recently emerged ST1193. Strains from both ST131-H30 and ST1193 carry three identical mutations—S83L and D87N in GyrA and S80I in ParC—the canonical signature of the FQR phenotype in *E. coli* that, with only minor modifications (usually in D87), is found in all other major FQR clones as well (Tchesnokova et al., 2019; Pitout et al., 2022). A recent study conducted in the Seattle area compared FQR *E. coli* in fecal samples obtained in 2015 and 2021 under the same collection protocol from women aged 50 and above who had not taken antibiotics for any reason for at least 12 months before providing the fecal sample (Tchesnokova et al., 2023). Surprisingly, while fluoroquinolone prescriptions decreased significantly in the local community between 2015 and 2021, there was a significant rise in the overall gut carriage of FQR *E. coli*. Especially notable was the rise of ST1193, which reached ST131-H30 in carriage frequency, and of FQR strains belonging to ST69, also carrying S83L in GyrA and S80I in ParC.

While Tchesnokova et al. (2023) provided the strongest evidence to date that carriage of MDR *E. coli* strains with certain QRDR mutations can occur and even expand without fluoroquinolone exposure, numerous earlier and subsequent studies support the sustained community circulation of FQR *E. coli*. Considerable colonization rates were reported for the ST131-H30 lineage and/or *E. coli* strains from diverse sequence types harboring QRDR mutations (Pallecchi et al., 2012; Zhong et al., 2015; Overdevest et al., 2016; Morales Barroso et al., 2018; Massella et al., 2020; Massella et al., 2021; Vianello et al., 2021; Zhao et al., 2021; Liu X. et al., 2022; Cherubini et al., 2022; Yap et al., 2024). Substantial incidence of ST1193 specifically among colonizing isolates was also observed (Zhao et al., 2021; Cherubini et al., 2022). Intriguingly, some of these studies were performed in settings with very low antibiotic selective pressure, such as the Amazon basin, yet still reported FQR *E. coli* with QRDR mutations colonizing healthy individuals (Pallecchi et al., 2012; Vianello et al., 2021; Yap et al., 2024). Moreover, multiple studies reported carriage of FQR *E. coli* harboring GyrA S83 and ParC S80 QRDR mutations in healthy children or in children about to be admitted to hospital—who would not typically be prescribed these antibiotics (Pons et al., 2014; Saksena et al., 2018; Singh et al., 2019; Cheng et al., 2022; Kibwana et al., 2023). Furthermore, *E. coli* strains harboring QRDR mutations, including ST131 and ST1193 isolates, were also cultured from community wastewater (Gomi et al., 2024). Human-to-human transmission as the primary mode of circulation for ESBL-producing ST131 and other FQR clones was reported by Verschuuren et al. (2026).

Two studies highlighted the longitudinal increase of community carriage of FQR strains. Marin et al. (2022) demonstrated that the FQR phenotype increased substantially among commensal *E. coli* strains carried by healthy French individuals between 2001 and 2010, even though no major shift in community quinolone use was observed during the study period (Bernier et al., 2014). A more than doubled rate of FQR *E. coli* carriage—dominated by ST131-H30 and QRDR mutation-carrying ST38—was observed between 2012–2013 and 2021–2022 among healthy children with no fluoroquinolone exposure in the Russian Federation (Suzhaeva et al., 2026).

Taken together, the available surveillance data on *E. coli* suggest that healthy individuals who have not been treated with fluoroquinolones for a long time, or ever, are commonly colonized with MDR strains belonging to globally disseminated FQR clones. Almost universally, these lineages carry QRDR mutations targeting residues S83 in GyrA and S80 in ParC. It is well established that gut-colonizing commensal-like *E. coli* are the primary source of ExPEC strains that cause urinary tract infections, bacteremia, meningitis, and other extraintestinal infections in the same individuals (Magruder et al., 2019; Denkel et al., 2020; Choi et al., 2024; Hong et al., 2024).

### 2.2 S. aureus

MRSA colonization in healthy humans and community spread has been reported by several groups (Huang et al., 2006; David and Daum, 2010; Kadariya et al., 2019; Cuny et al., 2024). It is well documented that the acquisition and extensive dissemination of MRSA strains—most prominently the community-associated USA300 (ST8) clone—was linked to substitutions at GyrA S84 and ParC S80 within the QRDR, with this sub-clade defined as USA300 North America (Muder et al., 1991; Panlilio et al., 1992; Alam et al., 2015; Lee et al., 2017; Challagundla et al., 2018a). During a period of more extensive fluoroquinolone use in the United States, ST8 strains harboring the double-serine mutations successfully invaded the healthcare setting and became the dominant healthcare-associated MRSA (HA-MRSA) lineage in the country (Planet, 2017; Challagundla et al., 2018a). Concurrently, this ST8 lineage demonstrated global significance, spreading to diverse geographic regions (Glaser et al., 2016). While a decline in fluoroquinolone consumption was associated with a reduced incidence of ST8 infections in US hospitals (Planet, 2017), numerous studies show that the QRDR mutations likely contributed to the successful community dissemination of ST8 isolates. The ST8 clone assumed the role of an important community-associated (CA) MRSA lineage, colonizing healthy individuals and causing community-acquired infections in the United States (Knox et al., 2012; Miko et al., 2012; Uhlemann et al., 2012; Uhlemann et al., 2014; Lee et al., 2017; Challagundla et al., 2018b) and in additional countries (Vindel et al., 2014; Manyahi et al., 2021; Tkadlec et al., 2023; Nyasinga et al., 2024).

Although some of these groups did not examine QRDR mutations, other studies clearly showed a high incidence of mutations at GyrA S84 and ParC S80, arguing for their role in facilitating community dissemination of the ST8 clone, which is well established as a cause of domestic infections. Uhlemann et al. (2012) emphasized that, among ST8 strains colonizing healthy individuals, those carrying the double-serine mutations proved to be “persisting” colonizers. The same group subsequently established that QRDR mutations were common in ST8 CA-MRSA strains isolated in New York and observed that these mutations may have “promoted the expansion” of the clone (Uhlemann et al., 2014). Alam et al. (2015) reported that the double-serine mutations were predominant (exceeding 80%) in ST8 CA-MRSA strains from New York, Los Angeles, and San Diego, and comprised 36% of isolates in Chicago. Lee et al. (2017) showed that CA-MRSA strains colonizing or infecting community patients were “differentiated by a stable chromosomal mutation in GyrA” and suggested that this genetic trait contributed to their dissemination. Challagundla et al. (2018a) observed that the GyrA S84 substitution was common in ST8 MRSA strains isolated from skin and soft tissue community-acquired infections and suggested that the genetic change could have facilitated “potential positive selection.”

As with the ST8 lineage, the double-serine mutations were reported to promote community dissemination of additional MRSA clones. Zhou et al. (2022) demonstrated that ST22 strains harboring double-serine mutations were prevalent in the community and have been moving into the healthcare setting in recent years in China. Sun Z. et al. (2025) observed that isolates from CC5, CC22, and other clones were common in outpatient ear infections, and the double-serine mutations were carried by most of these CA-MRSA strains regardless of sequence type. ST22 CA-MRSA strains harboring GyrA S84 mutations and moving into hospitals from the community were reported from both Portugal (Ferreira et al., 2021) and Pakistan (Ullah et al., 2022). Most recently, CC5 and CC22 CA-MRSA strains, many carrying at least one QRDR mutation, were shown to cause soft tissue infections in community-dwelling children in Spain (García-Cobos et al., 2025). The observation by Chen B. et al. (2022) that fluoroquinolone use increases the rate of nasal MRSA colonization, together with the report by Young et al. (2021) on an association between *S. aureus* colonization carrying the GyrA S84L substitution in the absence of fluoroquinolone exposure, serve as strong circumstantial evidence for the role of QRDR mutations in promoting colonization.

However, QRDR mutations are not the sole determinants of CA-MRSA colonization. ST22 strains of CA-MRSA carried by healthy individuals in the Gaza Strip were reported to lack *gyrA* or *parC* mutations (Chang et al., 2018), and the features responsible for the dissemination of these isolates remain to be identified. ST59 MRSA strains have extensively disseminated in both community and healthcare settings in some East Asian countries (McClure et al., 2021). The rate of QRDR mutations in the ST59 CA-MRSA lineage was reported to be low (Lai et al., 2018). The mutation rate affecting ParC S80 in ST59 MRSA strains isolated from healthcare settings in China was approximately 25%, and the substitution rate for GyrA S84 was reported to be very low (Wang et al., 2022). Accordingly, QRDR mutations did not play a major role in the dissemination of these ST59 strains. The spread of this lineage in the healthcare setting may have been facilitated partly by another fitness advantage: a much smaller antibiotic resistance gene cargo relative to the HA-MRSA lineages they replaced (Song et al., 2020; Chen et al., 2021; Wang et al., 2022).

Collectively, the available data suggest that many, although not all, MRSA strains from a variety of lineages retain one or two QRDR mutations even when residing in a low-fluoroquinolone environment such as the community. Although most of the studies mentioned above investigated community-acquired infections rather than colonization, most of these MRSA infections likely arose from prior colonization by the pathogens.

### 2.3 Clostridioides difficile

The *C. difficile* genome lacks the topoisomerase IV gene (Dridi et al., 2002); consequently, QRDR mutations conferring fluoroquinolone resistance reside in the *gyrA* and *gyrB* sequences, with the GyrA Thr82 mutation being equivalent to S83 in *E. coli*. Data on the colonization of healthy individuals with *C. difficile* carrying GyrA Thr82 substitutions remain less abundant, though increasing over the last decade. One of the earliest relevant studies was conducted by Ackermann et al. (2003), who, based on epidemiological observations, concluded more than 20 years ago that colonization with *C. difficile* harboring GyrA Thr82 mutations “may not be rare.” He et al. (2013) showed that the dissemination of the dominant global ST1 (ribotype 027) clone of *C. difficile* occurred “through at least two FQR lineages with clinical outcomes resulting in longer colonization duration.” Tian et al. (2016) reported that ciprofloxacin resistance rates were notably high among *C. difficile* strains isolated from healthy individuals in China. Abu-Khader et al. (2017) demonstrated that 40.5% of *C. difficile* strains cultured from asymptomatic Jordanian children not receiving any antibiotic treatment harbored *gyrA* and *gyrB* mutations. Williamson et al. (2019) observed that ST1 *C. difficile* strains carrying GyrA Thr82 mutations were common colonizers among healthy individuals.

The proper evaluation of *C. difficile* colonization data is complicated by the recent discovery that many strains colonizing healthy individuals are of zoonotic origin—a source to which many people may be regularly exposed (Mitchell et al., 2022; Lim and Riley, 2025). Since spore-forming bacteria possess a unique capacity to persist in diverse environments, the chances of transmission are substantial (Cersosimo et al., 2024). In addition, colonization by *C. difficile* is influenced by diet (Fishbein et al., 2022; Hewlett et al., 2025), intestinal inflammation (Barron et al., 2022), and certain virulence factors (Buddle and Fagan, 2023).

### 2.4 Additional MDR pathogens

Data on the carriage of additional MDR pathogens harboring QRDR mutations is also available. Multiple studies have documented carriage of FQR *Enterocococcus* faecium in healthy individuals or in patients with unrelated conditions. Novais et al. (2006) studied 99 healthy individuals in Portugal and isolated 96 *E. faecium* strains, 62.5% of which showed resistance to ciprofloxacin. Asadian et al. (2016) reported that 3.29% of *E. faecium* strains isolated from healthy individuals in Iran displayed ciprofloxacin resistance. Adesida et al. (2017) isolated 36 *E. faecium* strains from 100 healthy volunteers in Nigeria, of which 44.4% proved resistant to ofloxacin. Rios et al. (2020) characterized 12 vancomycin-resistant *E. faecium* strains from healthy individuals in Latin America, all of which were resistant to fluoroquinolones and carried QRDR mutations in positions equivalent to GyrA S83 and ParC S80 in *E. coli*. Mubarak et al. (2024) investigated 120 outpatients with gastroenteritis in Egypt, from whom 13 MDR *E. faecium* strains were cultured. Almeida-Santos et al. (2025) recently observed a marked decline in the prevalence of FQR *E. faecium* in healthy individuals in Portugal between 2001 and 2021.

Some data are available on the carriage of FQR *Klebsiella pneumoniae* in healthy individuals. Lin et al. (2016) observed that 28% of *K. pneumoniae* strains isolated from community-acquired urinary tract infections showed ciprofloxacin resistance and suggested that a considerable colonization rate was the most probable source of infection. Huynh et al. (2020) tested healthy pregnant women and demonstrated a 5% prevalence of colonization with FQR *K. pneumoniae*. Kader and Kamath (2009) found an incidence of less than 2% in Saudi Arabia, while Raffelsberger et al. (2021) did not detect any FQR *K. pneumoniae* in a study involving healthy Norwegian volunteers. In another Norwegian study, however, FQR *K. pneumoniae* was cultured from 9.9% of 284 healthy volunteers (Ulstad et al., 2016). Blaikie et al. (2024) found a 69% ciprofloxacin resistance rate in *K. pneumoniae* from healthy residents and wastewater of an aged care facility in Ethiopia.

The varied results for colonization rates of FQR *K. pneumoniae* and *E. faecium* may partly reflect differences in antibiotic exposure across study populations and methodological heterogeneity. The available data on colonization by *K. pneumoniae* harboring QRDR mutations in healthy individuals would warrant a comprehensive investigation.

Finally, a recent study demonstrated a high rate of levofloxacin resistance in *P. aeruginosa* strains carried by healthy individuals in China (Hu et al., 2023). No carriage data on MDR *P. aeruginosa* harboring QRDR mutations have been published to date.

## 3 Possible reasons for the association between QRDR mutations, MDR phenotype, and commensal success

The precise mechanisms underlying the ability of FQR lineages to demonstrate, on one hand, the MDR phenotype advantageous to pathogens and, on the other hand, high colonization fitness advantageous to commensals, remain to be established. Here, we review several potential explanations.

### 3.1 ‘Guilty by association’

It is possible that QRDR mutations are linked to the MDR phenotype simply by their prevalence in strains of clinical origin, rather than through any direct contribution to fitness. Fluoroquinolones have been, especially in the recent past, among the most frequently used antibiotics globally (Hicks et al., 2013; Jones et al., 2025; Kibwana et al., 2025; Madaras-Kelly et al., 2026). Historically, they were also the first-line choice for both the treatment and prevention of travelers’ diarrhea (Adler et al., 2022). Given this high selective pressure, the rise in resistance is unsurprising. This resistance often leads to treatment failure, prompting a switch to different, often broader-spectrum antibiotics, and consequently, various resistance markers accumulate within the genetic background of QRDR mutation carriers, leading to the emergence of MDR strains. While this scenario is plausible, it fails to explain why only specific clonal lineages become MDR and why they demonstrate increased competitive fitness.

### 3.2 Recombinational acquisition of QRDR mutations and improved allelic content

Evidence suggests that sets of multiple QRDR mutations can be acquired simultaneously through homologous recombination of GyrA and ParC and are responsible for the FQR phenotype acquisition of at least two major *E. coli* clones—ST1193 and ST410 (Tchesnokova et al., 2019; Chen L. et al., 2022). Such recombination events can result in the exchange of large chromosomal segments beyond the QRDR, leading to numerous allelic replacements, and may be driven by F(−like) plasmids, conjugative transposons, or integrative and conjugative elements (ICEs) (Hayes, 1953; Ochman et al., 2000; Feil et al., 2001; Dionisio et al., 2002; Frost et al., 2005). These exchanges can be highly beneficial (Kanesaka et al., 2022), helping the bacterial population purge its genome of deleterious mutations that would otherwise accumulate under purifying selection—a process known as Muller’s ratchet (Igelbrink et al., 2024). By resetting the mutational load, these events may improve overall fitness and facilitate the emergence of highly successful clonal groups (Igelbrink et al., 2024). The role of recombination in the evolution of MRSA, including the USA300 clone, is well documented (Castillo-Ramírez et al., 2012; Holden et al., 2013; Planet et al., 2013; Planet, 2017). A recent publication also demonstrated an effective transfer of large (100 + kb) chromosomal regions between ExPEC strains driven by ICEs (Berruga-Fernández et al., 2026). Thus, recombinational acquisition of the FQR phenotype could be accompanied by increased fitness through parallel exchange of other chromosomal regions. At the same time, it has been established that QRDR mutations can be acquired by successful clones in a stepwise manner that does not involve conjugative gene transfer (Hooper, 2000; Johnson et al., 2013).

### 3.3 Effect of QRDR mutations on plasmid stability

Another possibility is that GyrA and ParC alleles carrying QRDR mutations reduce the genetic conflict associated with the accumulation of large MDR plasmids. These plasmids often impose a mechanical burden on the cell, as they require constant resolution of DNA concatemers and management of supercoiling for stable inheritance (Zechiedrich et al., 2000). Mutations in type II topoisomerases may “fine-tune” global supercoiling density to a state that accommodates high plasmid loads with less metabolic friction (Bagel et al., 1999; Witz and Stasiak, 2010). By optimizing the topological environment, these QRDR mutations could lower the threshold for plasmid maintenance, essentially pre-adapting the lineage to carry extensive MDR gene cargo without a significant drop in stability. It is well established that plasmids can drive increased fitness of bacterial strains, including those with MDR phenotype (Ochman et al., 2000; Diep et al., 2006; Li et al., 2009; Pitout and Chen, 2023).

### 3.4 Co-evolution of virulence and colonization fitness

A recent study by Morel-Journel et al. (2025) involving longitudinal sampling of gut *E. coli* over many months to years in independent cohorts of adults (from the USA and France) demonstrated that *E. coli* strains can be divided into two types representing alternative fitness strategies. One type exhibited longer, subniche-specific gut persistence rate at relatively low abundance, while the other type showed a much higher colonization rate, also in a subniche-specific manner, with an ability to colonize the gut efficiently but in a more transient fashion. The persisting strains belonged to the same phylogenetic groups as the most successful FQR lineages—certain subgroups of the major phylogroups B2 and D—which also dominate among ExPEC isolates. ExPEC virulence factors such as bacterial cell wall-associated protectins, host cell-binding adhesins, and iron acquisition systems were strongly associated with the ability for long gut persistence. A higher prevalence of these virulence factors among resident versus transient *E. coli* strains in the intestines of healthy subjects was observed earlier (Nowrouzian et al., 2001), in accordance with the concept that many colonization factors also serve as virulence factors.

It is possible that, because antibiotic-resistant strains tend to persist longer in the virulence niche due to frequent failure of initial treatment regimens, their virulence properties may become better adapted for prolonged persistence in normally protected extraintestinal compartments, which in turn could confer a gut colonization or transmission advantage. Indeed, both ST131-H30 and ST1193 strains asymptomatically persist in the bladder more often than other clones (Tchesnokova et al., 2020), potentially helping them withstand elimination from the gut by occasional phages or highly competitive transient colonizers, allowing gut re-colonization and thus persistence. Besides *E. coli*, the USA300 clone of MRSA shows a combined, and potentially interconnected, ability to both cause disease and colonize or be transmitted among healthy carriers (Miller et al., 2012; Tong et al., 2015). However, further investigation is required to determine the extent to which the virulence of MDR clonal groups contributes to their commensal success and global spread.

### 3.5 Direct fitness effect of QRDR mutations

Multiple studies have shown that, despite altering proteins with an essential function across all prokaryotes, the canonical QRDR mutations do not confer a detectable fitness disadvantage in the absence of ciprofloxacin (Marcusson et al., 2009; Huseby et al., 2017). In the absence of a fitness cost, even occasional antibiotic use should provide a long-term advantage for community spread. However, another possibility—one we believe warrants special and expanded attention—is that certain QRDR mutations or their combinations are actually energetically favorable (Fuzi et al., 2017; Fuzi and Sokurenko, 2023). In other words, these mutations do more than shield bacteria from quinolones; they fundamentally alter the bacterial “energy budget,” providing a fitness windfall that manifests as accelerated growth rates and enhanced mucosal persistence. The hypothesis that additional fitness benefits facilitate MDR dissemination has been proposed by multiple groups for more than two decades (Martínez and Baquero, 2002; Shin and Ko, 2015; Bartke et al., 2021; Baquero et al., 2021; Dunn et al., 2021).

It has been hypothesized that the wild-type serine residues at positions GyrA 83 and ParC 80, which are highly conserved, are not functionally optimized but instead act as a chemical buffer against natural toxins produced by environmental competitors such as Streptomyces or various plant alkaloids (Hiramatsu et al., 2012; Morimoto et al., 2015; Morimoto et al., 2023). While these residues were protective in the pristine soil environments of the pre-antibiotic era, such toxins may be largely absent in the modern human gut and environment. When a pathogen like *E. coli* substitutes these serine residues with leucine or isoleucine, it may optimize topoisomerase function for the current environment, enabling faster replication. We hypothesize that this is the biological basis of the “fitness windfall.” Because MDR bacteria carry additional genetic cargo that typically imposes a high fitness cost (Andersson and Hugnes, 2010; Melnyk et al., 2015; Vanacker et al., 2023), QRDR mutations may provide the extra energy required to offset the costs of carrying and expressing an additional gene load without requiring a compensatory increase in metabolic activity.

Experimental findings strongly support the premise that positive fitness effects exerted by certain QRDR mutations make a significant contribution to the success of major international MDR clones. MRSA strains from major clones carrying favorable QRDR mutations were shown to propagate faster than isolates from minor clones that were unable to acquire these alterations (Horváth et al., 2012), findings fully consistent with clonal shifts observed in MRSA in Hungary during the preceding decade (Fuzi, 2016). Identical results were reported by a British group investigating clonal dynamics of MRSA in a British facility (Knight et al., 2012). Subsequently, strains from major international clones of MDR *K. pneumoniae* also showed significantly faster growth rates relative to minor clone isolates unable to acquire favorable mutations—fully in line with clonal dynamics in the country (Toth et al., 2014).

Although not yet definitive, the evidence provides a strong rationale for expanding studies in this direction. It is possible that the fitness benefit conferred by the energetically favorable “double serine” mutations is realized only in certain natural variants of GyrA and ParC, in specific clonal genomic backgrounds, or in particular hosts, potentially depending on multiple underlying conditions including diet. If proven, the concept of a direct fitness benefit from QRDR mutations would provide the most compelling argument for the colonization success of major MDR clonal lineages and suggest novel approaches for reducing their burden.

## 4 Lack of a strong animal connection

The question arises as to how much the high carriage rate of successful MDR clonal lineages harboring *gyrA* and/or *parC* mutations in healthy individuals can be linked to transmission from the veterinary sphere.

It is well established that, as a consequence of nalidixic acid and enrofloxacin use, quinolone resistance is not rare in *E. coli* isolated from livestock and retail food products (Hayer et al., 2020; Nguyen et al. Mounsey et al., 2021; Li et al., 2022; Gelalcha et al., 2023; Soncini et al., 2024; Sun J. et al., 2025). Human transmission events inevitably occur. However, the incidence of the major international MDR lineages isolated from livestock is surprisingly low. Only a few papers reported detection of ST1193 strains in animal husbandry and the retail industry (Watt et al., 2025; Dantas et al., 2025), but these likely stem from downstream cross-contamination during food processing. In contrast, a large number of studies found no ST1193 strains in the veterinary sphere, arguing strongly for a non-veterinary origin of most human commensal ST1193 isolates (Johnson et al., 2017; Umpiérrez et al., 2017; Hayer et al., 2020; Nguyen et al., 2021; Kim et al., 2021; Wang et al., 2021; Cox et al., 2022; Johnson et al., 2022; Li et al., 2022; Benlabidi et al., 2023; Kravik et al., 2023; Gelalcha et al., 2023; Mwenifumbo et al., 2023; Soncini et al., 2024; Aziz et al., 2024; Kuan et al., 2024; Masarikova et al., 2025; Tian et al., 2025; Zhu et al., 2026; Klaas et al., 2026).

The prevalence of the ST131 lineage is somewhat greater in animal and retail samples, although in most cases it was either not investigated or not confirmed to belong to the pandemic FQR subclone H30. Multiple groups demonstrated the presence of ST131 *E. coli* strains in various animal and retail products (Johnson et al., 2017; Liu et al., 2018; Johnson et al., 2022; Runcharoon et al., 2025; Klaas et al., 2026) although sometimes only in low numbers (Hayer et al., 2020; Cox et al., 2022; Aziz et al., 2024; Soncini et al., 2024). Alternatively, numerous groups investigating the livestock and retail sphere did not detect any ST131 strains (Umpiérrez et al., 2017; Röderova et al., 2017; Nguyen et al., 2021; Kim et al., 2021; Wang et al., 2021; Li et al., 2022; Benlabidi et al., 2023; Kravik et al., 2023; Gelalcha et al., 2023; Mwenifumbo et al., 2023; Kuan et al., 2024; Masarikova et al., 2025; Tian et al., 2025; Zhu et al., 2026). Some ST131 reports noted that the sub-lineages identified were not related to the FQR clone ST131-H30 (Johnson et al., 2017; Liu et al., 2018). Runcharoon et al. (2025) showed that only some isolates showed overlap between human and chicken samples. These findings suggest a limited, rather than substantial, epidemiological relationship. At least one study reported isolation of ST131-H30 subclones in retail poultry, but with no evidence that this originated from livestock rather than from meat handling (Ghodousi et al., 2016).

Another *E. coli* clonal group with FQR/MDR sub-clones, ST69, was more frequently isolated from animal species and has been detected in retail products (Umpiérrez et al., 2017; Hayer et al., 2020; Kim et al., 2021; Johnson et al., 2022; Li et al., 2022; Benlabidi et al., 2023; Gelalcha et al., 2023; Kravik et al., 2023; Mwenifumbo et al., 2023; Kuan et al., 2024; Aziz et al., 2024; Masarikova et al., 2025; Tian et al., 2025; Klaas et al., 2026; Zhu et al., 2026). However, it remains unclear whether human-associated FQR sub-lineages are part of the ST69 isolated from livestock or retail products, and multiple groups did not detect the ST69 lineage in similar samples (Nguyen et al., 2021; Wang et al., 2021; Cox et al., 2022; Soncini et al., 2024). Isolation of strains from MDR clonal groups ST410 and ST10 has also been reported (reviewed in Yartey et al., 2025), suggesting high fitness across different host species. Overall, however, there is a growing consensus in the field that MDR clonal groups of ExPEC from humans and livestock do not significantly overlap, suggesting that successful cross-species transmission events are rare or transient and questioning the view that farmed animals are a major reservoir for the dominant clonal groups of drug-resistant *E. coli* isolated from human infections (Singer, 2015; Cormier et al., 2019; Findlay et al., 2020; Mounsey et al., 2021). At the same time, horizontal transfer of genetic material via quinolone- and other resistance-encoding plasmids and conjugation-driven recombination is certainly possible between animal- and human-adapted strains and could potentially contribute to the emergence of MDR strains circulating in human communities (Cormier et al., 2019).

In contrast to farmed animals, ST131, ST1193, and ST69 lineage strains are frequently isolated from domestic pets, allowing for mutual transmission with humans (Salgado-Caxito et al., 2021; Habib et al., 2023; Watt et al., 2025; Maeda et al., 2025). Some studies reported high rates of FQR *E. coli* assigned to diverse sequence types in urban pets (Kidsley et al., 2020; Habib et al., 2023; Maeda et al., 2025), suggesting that, similarly to humans, carriage of these resistant isolates exists in pet animals, most likely as a result of spillover transmission. Similar to house pets, humans are most likely the primary source of the successful FQR/MDR clones isolated from human-associated environments such as municipal wastewater and treatment plant run-off, which in turn can serve as a source of contamination for wildlife and water-treated agricultural fields (Jamborova et al., 2018; Cabal et al., 2021; Puljko et al., 2024; reviewed in Kocsis et al., 2022).

The origin of MRSA strains from animals has also been studied in great detail. As with *E. coli*, the consensus is that the majority of MRSA clonal groups originated from within human populations, in hospitals or in the community. Human origin is strongly supported for USA300/ST8 and heavily FQR lineages ST5, ST22 (EMRSA-15), ST239, and ST36 (Enright et al., 2002; Van Boeckel et al., 2014; Mediavilla et al., 2012; Lakhundi and Zhang, 2018; Chambers and DeLeo, 2009). While a farmed animal origin (from pigs, cattle, or chickens) is established for ST398, ST9, and ST97, these are relatively rarely isolated in clinical settings and mostly colonize farm animal handlers and their households (De Neeling et al., 2007; Graveland et al., 2011; Van Boeckel et al., 2014; Cuny et al., 2015).

Animal origin of, or a sustained animal reservoir for, other MDR pathogens such as ST258 and other carbapenem-resistant *K. pneumoniae* is also questionable, given the rarity of isolation from livestock (Munoz-Price et al., 2013; Navon-Venezia et al., 2017; Pitout et al., 2015).

**Figure 1:**
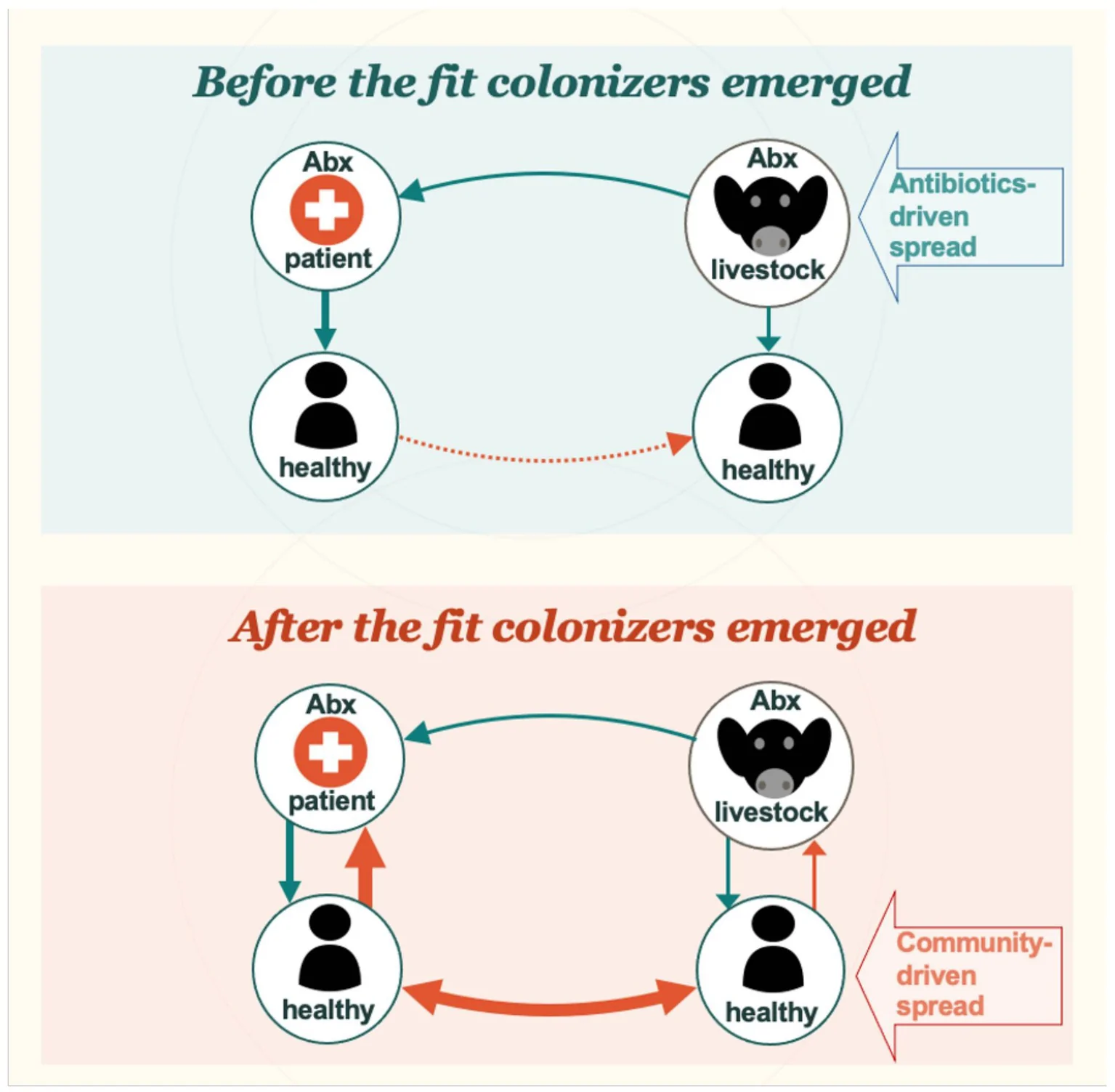
Circulation of antibiotic-resistant strains before and after the emergence of highly fit colonizing strains. Arrows represent the extent of transmission of Abx resistant bacterial strains. In blue: low fitness colonizers. In red: high fitness colonizers.

Thus, taken together, the studies summarized above imply that the primary mode of circulation for major FQR/MDR clonal lineages of human bacterial pathogens is colonization of and transmission between healthy individuals as part of the commensal microbiome. Antibiotic use in patients was undoubtedly critical for the emergence of the original resistant strains, likely with some contribution from gene flow or direct transmission of resistant strains from livestock. It is also plausible that at early stages their colonization fitness was reduced and contingent on antibiotic use, with transmission between healthy individuals in the community being unsustainable (Figure 1). As discussed above, the exact molecular mechanisms and evolutionary steps underlying the emergence of FQR strains with high commensal fitness remain unclear. However, once highly fit MDR strains emerged, their colonization and transmission between individuals became independent of—or only periodically aided by—antibiotic use (Figure 1). This in turn drove the emergence of highly successful clonal groups that became the major driver of the rise of antibiotic resistance and treatment failure in patients with both community- and hospital-acquired infections.

## 5 Strategies for the eradication of colonizing MDR pathogens

The recognition of the severity of this problem has prompted diverse endeavors to eradicate MDR bacterial pathogens from the commensal flora (reviewed in Roson-Calero et al., 2023). Diverse strategies have been tested; however, with the exception of fecal microbiome transplantation for *C. difficile* and effective disinfectants for MRSA, none has proved durable yet.

Initial attempts focused on the use of various antibiotics, but this approach was largely ineffective for intestinal colonizers (Buehlmann et al., 2011; Saidel-Odes et al., 2012; Huttner et al., 2013) and was shown to trigger adverse effects (Farinas et al., 2021). These efforts were largely abandoned, and an array of alternative strategies was devised developed.

As noted above, the most promising results have been obtained with fecal microbiome transplantation, primarily for the decolonization of *C. difficile* (Lee et al., 2016; Kelly et al., 2021; Ooijevaar et al., 2021). The technique is now strongly recommended for the eradication of both wild-type and MDR strains from the intestinal tract (Baydoun et al., 2025; Dinis et al., 2025).

Studies attempting decolonization of other MDR pathogens by fecal transplant have yielded mostly less compelling results (Huttner et al., 2019; Seong et al., 2020; Shin et al., 2022; Davido et al., 2024; Nooij et al., 2024; Rashidi et al., 2024; Kriz et al., 2024; Lin et al., 2025), although some groups reported promising findings in studies of limited scope (Bier et al., 2023; Woodworth et al., 2025). Nooij et al. (2024) recently noted that, while fecal transplants can be useful, they will not “fully fix the problem” of MDR pathogen eradication.

Decolonization of MDR pathogens from the gastrointestinal tract by phages has appeared to be a straightforward approach but is now under considerable scrutiny. Numerous papers studies in animal models have yielded highly variable results (Galtier et al., 2016; Javaudin et al., 2021; Porter et al., 2022; Fang et al., 2022; Liu J. Y. et al., 2022; Lourenço et al., 2023). Studies conducted in patients using phages and phage-antibiotic combinations yielded similarly varied outcomes (Kuipers et al., 2019; Corbellino et al., 2020). Several authors emphasized that a number of challenges must be addressed before phage therapy can be established as a routine treatment option (Roach and Debarbieux, 2017; Furfaro et al., 2018; Harper, 2018; Campos-Madueno et al., 2023).

Decolonization of MDR pathogens from the intestines by probiotics has also yielded mixed results (Dall et al., 2019; Ramos-Ramos et al., 2020; Ljungquist et al., 2020; Zollner-Schwetz et al., 2020; Wieers et al., 2020). However, the study by Morel-Journel et al. (2025) suggested that the best competitors of colonizing strains from MDR clonal groups could be closely related strains lacking the resistance phenotype. The success of this approach might be enhanced in combination with phage therapy administered in parallel, as suggested by a recent animal study (Porter et al., 2022).

Another approach is to use programmable CRISPR-Cas systems delivered to kill or disarm specific strains via polymer nanocomposites, microparticles, phages, or conjugative bacteria (Fagen et al., 2017). While some studies have yielded highly successful results (Neil et al., 2021), an efficient delivery system still needs to be developed.

The development of an effective and specific vaccine is hampered by the requirement to target multiple species while preserving the integrity of the intestinal commensal, non-resistant microbiome. However, considering that the major clonal groups of *E. coli* are characterized by a relatively uniform and specific set of vaccine-targetable surface components (LPS, capsules, outer membrane proteins, etc.), this approach is worth exploring further.

In conclusion, the issue of human intestinal colonization by MDR bacterial pathogens remains unresolved. A technique reliably eliminating major MDR clone strains without compromising the commensal gut microbiome remains to be developed.

The eradication of MRSA from the nasopharynx and skin of carriers is easier than decolonization in the gut and can be accomplished by relatively safe means—using either an appropriate topical antibiotic (Saberi et al., 2025) or an efficient disinfectant applied to the affected area (Qian et al., 2025). The success of mupirocin/chlorhexidine decolonization in preventing both hospital- and community-acquired infections is well documented (Van Rijen et al., 2008; Bode et al., 2010; Fritz et al., 2011; Climo et al., 2013; Huang et al., 2013; Septimus and Schweizer, 2016). However, rising resistance to decolonizing compounds may become a significant problem in the future (Robicsek et al., 2008).

## 6 Conclusion

We believe that the emerging evidence for commensal carriage of FQR/MDR pathogens in healthy individuals currently poses the greatest threat to public health, surpassing the ongoing threats of antibiotic use in patients or livestock and environmental contamination. It is the colonization-to-infection transition in carriers, and transmission from individuals with robust immunity to those with a compromised immune state, that represents the main source of drug-resistant clinical infections. The key problems are that the community serves as the reservoir for antibiotic-resistant strains and that surveillance at this level remains severely lacking. Unfortunately, efforts aimed at developing efficient measures for eliminating these resistant pathogens from the normal intestinal flora have not yielded a reliable approach to date. Further studies are needed to elucidate the true rate of intestinal colonization by these resistant pathogens, and efforts must be greatly intensified to find a reliable way of their eradication.
